# Effectiveness of an intervention to improve supportive care for family caregivers of patients with lung cancer: study protocol for a randomized controlled trial

**DOI:** 10.1186/s13063-017-2044-y

**Published:** 2017-07-04

**Authors:** Michèle Aubin, Lucie Vézina, René Verreault, Sébastien Simard, Jean-François Desbiens, Lise Tremblay, Serge Dumont, Lise Fillion, Maman Joyce Dogba, Pierre Gagnon

**Affiliations:** 1 Research Centre of the Institut Universitaire de Cardiologie et de Pneumologie de Quebec (IUCPQ), Quebec, QC Canada; 2 Research Centre of Primary Care and Services-Université Laval (CERSSPL-UL), Quebec, QC Canada; 3 Research Centre of the CHU de Quebec, Quebec, QC Canada; 4 Department of Family Medicine and Emergency Medicine, Université Laval, Quebec, QC Canada; 5 Maison Michel-Sarrazin Research Team in Palliative Care and Psychosocial Oncology (ERMOS), Quebec, QC Canada; 6 Laval Family Medicine Unit, Université Laval, Quebec, QC Canada; 7 Department of Social and Preventive Medicine, Université Laval, Quebec, QC Canada; 8 Pulmonary Oncology Centre, IUCPQ, Quebec, QC Canada; 9 Faculty of Nursing, Université Laval, Quebec, QC Canada; 10 Department of Medicine, Université Laval, Quebec, QC Canada; 11 Faculty of Social Sciences, Université Laval, Quebec, QC Canada; 12 Department of Psychiatry, Université Laval, Quebec, QC Canada; 13 CIUSSS Capitale-Nationale , 2690, Chemin des Quatre-Bourgeois, Quebec, QC G1V 0B7 Canada

**Keywords:** Randomized controlled trial, Lung cancer, Family caregivers, Supportive care

## Abstract

**Background:**

Family caregivers (FC) often experience higher distress levels than their relative with cancer. Many cancer centers have implemented distress screening programs, but most of them concentrate their efforts on patients, with little attention to their FC. To fill this gap, a pragmatic intervention has been designed to improve supportive care for FC of patients with lung cancer. This article describes the study protocol of a single-center randomized controlled trial to assess its effectiveness.

**Methods/design:**

A total of 120 lung cancer patients and their FC are randomly assigned to the experimental group (exposed to intervention, *N* = 60) or to the control group (usual care, *N* = 60). The intervention includes: (1) systematic FC distress screening and problem assessment near their relative’s cancer diagnosis, and every 2 months, (2) privileged contact with an oncology nurse (ON) away from the patient to address FC problems and (3) liaison by the ON with the family physician of FC reporting high distress (thermometer score ≥5/10), or problems relying on FP expertise. In both groups, FC, patient and process-of-care outcomes are measured at baseline and every 3 months, up to 9 months. The primary endpoint is FC distress measured by the Hospital Anxiety and Depression Scale (HADS) and the Psychological Distress Index used in the Quebec Health Survey (PDQHS). Individual interviews with 10 FC and a focus group with the oncology team will be conducted at the study end to further document the effectiveness of the intervention and its impact on quality of life (for FC) and practice organization (for the oncology team).

**Discussion:**

This trial will assess the effectiveness of an innovative intervention based on interprofessional collaboration between primary care and oncology care. It targets a population in great need, yet often neglected, and has the potential to clearly improve patient and caregiver experience of cancer care, and reduce the burden of disease.

**Trial registration:**

ClinicalTrials.gov, ID: NCT02531464. Registered on 15 July 2015.

**Electronic supplementary material:**

The online version of this article (doi:10.1186/s13063-017-2044-y) contains supplementary material, which is available to authorized users.

## Background

A diagnosis of cancer is emotionally threatening not only for patients but also for their family caregivers (FC) who witness and share much of the illness experience of their relative [1]. Along the cancer care trajectory, FC play a critical role in providing ongoing homecare and assistance to their loved one [1]. However, they are often unprepared to play their role and may experience growing levels of distress, as their relative’s condition deteriorates. Even though many FC are coping quite well, studies show that 10 to 50% of them report high distress [2, 3] which is often more severe than in patients [2, 4–7], and it may persist over time, with a potential impact on their physical, emotional and social health [1, 4, 8]. FC of lung cancer patients are particularly vulnerable [7] and likely to be distressed [9–13] because of a rapid evolution [2, 8, 14] and decline in their relative’s functional status associated with symptoms and treatment side effects [1, 15–17].

FC may often feel unsupported in their role [18] and would need more information and tips to fulfill it [18]. Their unmet needs may compromise their quality of life (QoL) [19] and contribute to their distress [20], but also they may adversely impact on their relative’s distress [21]. Because of the interrelation of the patient-FC dyad experience, identifying and meeting FC needs should be considered as an integral part of cancer services [22–25]. It is recommended to assess FC distress and needs early after diagnosis, and to reassess them regularly with cancer progression [26, 27] in order to support them and help them play their role in patient assistance and ongoing care throughout the care trajectory. However, little is known on the most effective interventions to develop and how to implement them into routine practice [13, 27].

As recommended by many expert groups and health authorities around the world [22–25], many cancer centers have implemented distress screening programs. However, most of them concentrate their efforts mainly on patients. In order to fill this gap, an intervention was developed to improve supportive care for FC.

### Development of the intervention

**Fig. 1:**
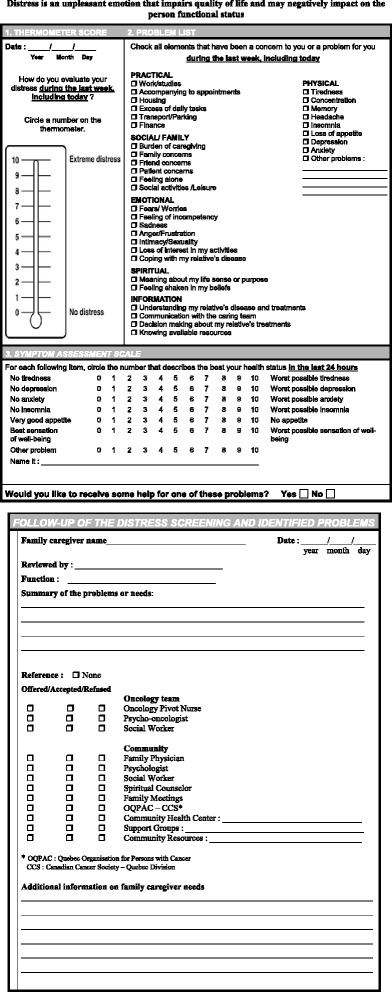
Distress Screening tool adapted to the family caregiver (FC) context

The multifaceted intervention was developed according to the knowledge gained from the academic literature, from previous research [28] and from recommendations of expert groups and health authorities [22–25]. The Screening for Distress tool currently used for cancer patients in Quebec, Canada (including the Distress Thermometer with the Canadian Problem Checklist and the Edmonton Symptom Assessment Scale [29, 30]) was adapted to the FC context (Fig. 1). Then, a pilot study was conducted, consisting in individual interviews with 12 FC of patients at different phases of their lung cancer (near diagnosis, during chemo/radiation therapy, after the end of treatments), to get their comments and impressions regarding this tool and the other components of the intervention. FC also gave their opinion on the most appropriate timing and frequency for completing the Screening for Distress tool, and were invited to suggest any other strategies that they would consider useful to fulfill their needs. In addition, a focus group was conducted with members of the IUCPQ oncology team and their decision-makers, (where the intervention is tested), to ensure its acceptability and feasibility in their practice. IUCPQ is a tertiary care center in cardiology and pulmonology in Quebec City (Quebec, Canada) where more than 700 new patients with lung cancer are followed each year. The oncology team (OT) is composed of pulmonologists responsible for the treatment of these patients, oncology pivot nurses (OPN) who play a role of resource person and cancer navigator for patients and their family (each patient has a designated OPN to refer to whenever needed), oncology nurses, social workers, a psycho-oncologist, pharmacists and nutritionists. Finally, a focus group with eight family physicians (FP) from different community-based practice settings (private clinics, family medicine teaching units, community health centers) was conducted to ensure that the intervention was also acceptable and feasible in their practice.

With the agreement of participants, individual interviews and focus group discussions were recorded to facilitate data collection. A content analysis of the recordings was performed independently by a research professional and one researcher to identify the modifications to the intervention that were suggested by the different participants. They compared their notes and jointly produced a summary discussed with the research team that served to adapt and refine the multifaceted intervention accordingly.

## Objectives

The main objective of this study is to implement and assess the effectiveness of an intervention integrating community-based primary care with oncology care to improve supportive care for FC of lung cancer patients. More specifically, this trial aims to assess the effectiveness of the intervention on: (1) *FC outcomes*: distress (primary outcome), anxiety, depression, quality of life (QoL), needs, burden, perception of health, preparedness in caregiving, social support, care satisfaction, (2) *Patient outcomes*: distress, anxiety, depression, QoL, pain/other symptom relief, (3) *Care process outcomes*: FC and patient utilization of services. In addition, a qualitative component of the study will further document, in the experimental group, FC-perceived usefulness of the intervention and its effects on distress/QoL, and in the oncology team, the perceived usefulness of the intervention and its effects on their practice and organization of care.

## Methods/design

**Fig. 2:**
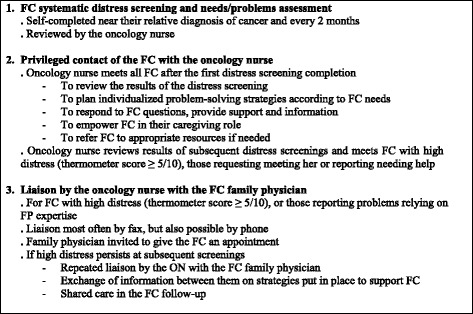
The multi-faceted intervention

A two arm-randomized controlled trial is conducted at a single center, the IUCPQ Pulmonary Oncology Clinic, to assess the effectiveness of the intervention (Fig. 2).

### Description of the intervention

In the current practice at the IUCPQ Oncology Clinic, there is a systematic distress screening program for patients, but not for FC. In their role towards patients and their family throughout the cancer care trajectory, may provide some support and information to FC and, in rare occasions, refer them to appropriate resources. However, most of their interventions target patients, and only few FC receive support services. There is no systematic distress screening and problem assessment for FC, nor any standardized service and resource specifically dedicated to them. The study intervention directly targets FC of patients with lung cancer and aims to increase the support provided to them. In order to prevent contamination of the control group, an oncology nurse (ON) with the training of an OPN [31, 32] has been recruited specifically to administer the intervention to FC randomly assigned to the experimental group. The ON intervenes in addition to the usual care provided by an OPN to patients and their family. The finalized intervention includes three components and is summarized in Fig. 2:

1. *Systematic distress screening and needs/problems assessment of family caregivers by the oncology nurse*
  FC from the experimental group complete the adapted Screening for Distress tool early after the diagnosis of their relative, and every 2 months during the 9-month study. Whenever possible, they complete this tool when they accompany their relative at a regular visit to the clinic. Otherwise, arrangements are made by the ON to either schedule a specific visit for FC distress screening or mail them a copy of the screening tool and complete it with them by phone. The ON reviews the results of the Screening for Distress tool
2. *Privileged contact of the family caregiver with the oncology nurse*
  The ON meets all FC away from their relative, after their first completion of the distress screening and needs/problems assessment tool. This professional discusses with them results of this tool, and further assesses their needs in order to plan with them individualized problem-solving strategies, and, if necessary, refers them to professional resources from the IUCPQ Oncology Clinic or the community. The training as an OPN provides the ON the basic skills to support FC, acknowledging that it is normal for them to go through different emotions (anxiety, fear, sadness, helplessness, etc) in their situation, and counsels them on the importance of taking care of them and preserving time for them, while accompanying their relative. The ON provides community resources and educational material (in addition to what is usually given) related to their caregiving role, and helps them to become better prepared and more proactive in their role, also answering all remaining questions asked by FC. Then, every 2 months, the ON reviews FC results of the screening tool and meets those who present a high level of distress (thermometer score ≥5/10) or who indicate needing help. The ON follows-up on prior strategies put in place to help FC and adjusts them to the current situation. So the ON acts as a resource person for FC throughout the follow-up in oncology
3. *Liaison by the oncology nurse with the FC family physician*
  For FC reporting high levels of distress (thermometer score ≥5/10) or experiencing problems relevant to FP expertise, the ON transfers to their FP, information on their identified needs and level of distress, and also provides to them information on any strategy put in place or reference to other health professionals to respond to FC needs. The ON invites FP to give an appointment to their patient, and facilitates a shared follow-up of the FC when needed. If FC continue to present high levels of distress or many needs/problems at subsequent screenings, the ON transfers the information to their FP and organizes another appointment/follow-up. Most communications between the ON and FP occur by fax, but they may also communicate by phone if needed.

### Study population

**Fig. 3:**
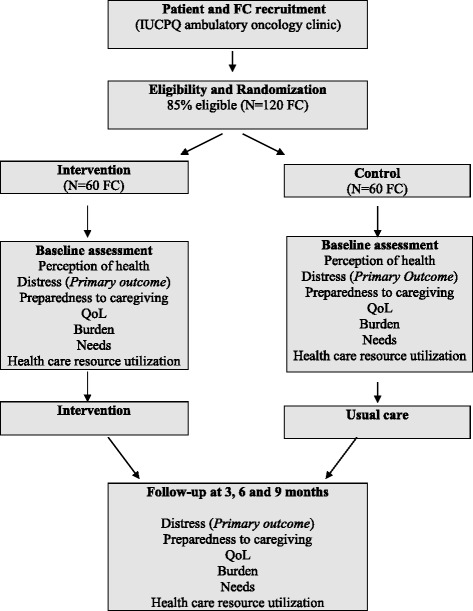
Trial flow diagram

Patients diagnosed with a nonsurgical lung cancer (corresponding to more than 85% of all cases), having an estimated life expectancy of at least 4 months (to minimally ensure two data collections), and with FC identified by patients as their main caregiver, and who report having a regular FP, are invited to participate to the study by the OPN. FC are eligible independently of their health status. Using a computer-generated randomization list, these dyads are randomly assigned by the research team to either the experimental group (exposed to the intervention) or to the control group (usual care), as shown in Fig. 3. As the study focuses mainly on FC, they can participate even if their relative with cancer decides not to participate or withdraws before the end of the study. There is no blinding in this trial, so participants and the research team know the group allocation.

As FC distress is the primary outcome in this study, the sample size was calculated from the mean scores of distress found in a prior study conducted by our team with a similar population of FC of patients with lung cancer (unpublished abstract presented at the 16th World Congress of Psycho-Oncology, Lisbon, Portugal, 23 October 2014). Based on FC distress scores of respectively 13.7 ± 7.0 at the Hospital Anxiety Depression Scale (HADS) (range = 0–42) and of 23 ± 16 at the Psychological Distress Scale used in the Quebec Health Survey (PDSQHS) (range = 0–100), a sample size of 120 FC (60 per group) was calculated. Considering a 5% *α* error, a power of 80% and an anticipated withdrawal rate of 20% (due to patient deterioration or death), this sample size allows to detect a 33% difference in distress scores between the two groups which is considered clinically significant.

### Procedures and data collection

At a regular visit of the patient who is most often accompanied by their FC, the OPN briefly explains the study to them and asks permission to transfer their phone number to the study team. Then, a research professional contacts them to present the study more extensively, get their agreement to participate and set an appointment with them to sign the Consent Form and begin the data collection with them. In both groups, the research professional interviews separately patients and FC at baseline and at 3-month intervals, for a maximum of 9 months, or until the patient’s death (maximum of four data collections), thus minimizing missing data (Fig. 3). Validated tools are used to collect all the study outcomes. At baseline, FC respond to basic questions related to their personal and medical characteristics. They also complete the following validated questionnaires:

- Primary outcomes:
  - *Distress* measured by two instruments: (1.1) the HADS [33, 34], a 14-item instrument specifically designed to evaluate distress, anxiety and depression. For each item, the scale varies from 0 (most of the time) to 3 (never). Using the standard criteria suggested by authors of this instrument, distress scores are converted into two categories (absence/presence of distress) while anxiety and depression scores are converted into three (absence/suspected/significant anxiety or depression); (1.2) the global score of the abbreviated version of the PDSQHS [35, 36]. This14-item instrument was derived from the Psychiatric Symptoms Index [37] and has been used in the Quebec general population to estimate the prevalence of clinically significant distress (scores ≥80th percentile), so it permits the comparison of proportions of FC with high distress levels to the ones found in the general population. The PDSQHS scale varies from 1 (never) to 4 (very often).
- Secondary outcomes:
  - *Needs*. The Home Caregiver Need Survey [38] is a 25-item tool covering four categories of needs (informational, practical, emotional and spiritual). FC indicate, for each need, its perceived importance, on a 0 (not important) to 4 (extremely important) scale, and how well it is satisfied (0 = unsatisfied to 4 = completely satisfied).
  - *Psychological burden*. The French version of the Caregiver Burden Scale in End of Life Care (CBS-EOLC) [39] is a 16-item instrument validated among FC who assist cancer patients in palliative phase. Items vary from 1 (never) to 4 (very often).
  - *Preparedness for caregiving*. The Preparedness for Caregiving Scale [40] measures the perceived readiness for multiple domains of the caregiving role (providing physical care, emotional support, setting up in-home support services and dealing with the stress of caregiving). This eight-item instrument uses a 0 (not at all prepared) to 4 (very well prepared) scale.
  - *Quality of Life*. The City of Hope-QoL Scale-Family Version [41] is a 37-item instrument measuring four QoL domains (physical, psychological, social and spiritual), with questions using a 0–10 scale.
  - *Service and health care resource utilization*. FC report the number of visits to their FP and to any other health/psychosocial professional, meetings to support groups, use of community resources (volunteer services, respite, help for housework, etc.), sick leave, and prescription of sleeping pills, anxiolytic or antidepressant medications.

**Fig. 4:**
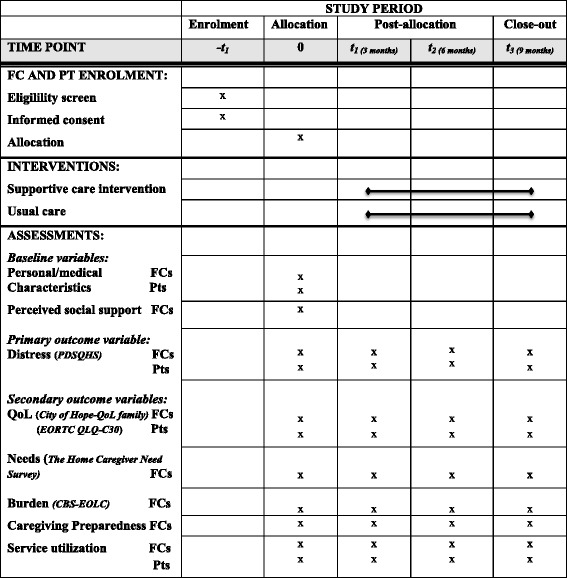
Schedule of enrollment, interventions and assessments. FC: family caregiver, Pts: patients

Measures are also taken at similar intervals with FC relatives with lung cancer, since their physical and mental health may influence FC. At baseline, patients respond to basic questions related to their personal and medical characteristics, and their functional status based on the Karnofsky scale [42] which has a well-known correspondence to the Eastern Collaborative Oncology Group (ECOG) scale [43]. As for FC, they complete distress measures (PDSQHS and HADS) every 3 months, a *Healthnnrelated QoL* measure (European Organization for Research and Treatment of Cancer Quality of Life Core 30: EORTC QLQ-C30) [44], and *service and health care resource utilization* (frequency of emergency room (ER) visits, hospitalizations, homecare services or other services from the community health center). Figure 4 shows the schedule of enrollment, interventions and assessments for all participants.

To monitor the administration of the intervention, additional information is collected with FC from the experimental group reporting high distress at screening: the communication with FP, strategies put in place and references to specific resources, as well as the number of FC visits or phone contacts.

In order to promote participant retention, data collections are usually scheduled on the day of a patient regular visit to the oncology clinic. If no visit is planned at the time of a data collection, the questionnaires are mailed to the participants and they are contacted by phone to determine the most convenient way to complete them (either by themselves or with the research professional who may go to their home or complete the questionnaires with them over the phone).

Data from the questionnaires are coded in two separate files and secured in a computer with a passcode. The computer is located in a locked room accessed only by the research team. A SPIRIT Checklist of all items recommended in a randomized controlled trial is included in Additional file 1.

Finally, individual interviews will be conducted with a subgroup of FC in the experimental group at the end of the study (*N* = 10 or until saturation) to document their perception of the intervention usefulness and effect on their QoL. Also, a focus group will be conducted with the OPN and others professionals of the IUCPQ OT, to assess their perception of the usefulness of the intervention and its effect on their practice and organization of care.

### Analysis

The quantitative analysis plan first includes an evaluation of the adherence to the planned intervention by verifying for each FC from the experimental group: (1) the proportion of planned screening tools actually completed, (2) the proportion of liaisons with FP, (3) a descriptive analysis of all interventions performed by the ON (number of encounters, referrals, provision of educational material, etc.). Then, FC primary and secondary outcomes will be compared between the experimental and control groups at each data collection period. In addition, mixed models for repeated measures will be constructed with FC, patient and process-of-care outcomes to assess the intervention effectiveness. These analyses of variance for repeated measures will compare the fluctuation over time of FC distress and other variables between the experimental and control groups. Whenever significant changes are found within one group for certain outcomes over time, the mixed models for repeated measures will help to identify when the change occurred. Finally, McNemar tests will be used to compare FC and patient distress.

The qualitative analysis will consist in content analysis of the recordings of FC individual interviews and the focus group with the OT, based on Miles and Huberman methodology [45], to further document the perceived usefulness of the intervention and its effect on FC QoL and on the OT practice and organization of care. Each interview will be independently analyzed by a research professional and one researcher to ensure interobserver reliability. In case of disagreement, perceptions will be reviewed until a consensus is reached.

## Discussion

FC play a crucial role in cancer patient care, representing their principal source of support [1]. However, they often have their own lives put into upheaval [2, 8, 13, 14] and neglect their health and needs to focus on supporting their relative with cancer [4, 46]. It is well recommended to provide FC with the resources, information and support needed to maintain good health, and to sustain their caregiving role [9, 27]. As emotional support appears to be an important part of their role in cancer care [47, 48], FP could be important actors in assessing FC health and advising them about resources to facilitate their caregiving role [49]. The long-standing relationship with their patients and their usual familiarity with patient social context put them in a strategic position to provide emotional support. It is, thus, reasonable to assume that FP may favorably influence FC distress when they accompany a relative diagnosed with cancer.

Systematic screening for distress programs has been implemented in many oncology centers, but their focus has been limited to cancer patients, with little intervention on FC. The intervention tested in this study aims to address this gap by targeting more particularly FC, a population in great need, yet often neglected. The intervention has been pilot-tested to ensure its acceptability for members of an oncology team working in a tertiary care hospital and for community-based FP, and also to confirm the appropriateness of the Screening for Distress tool, adapted to the FC context, as part of the intervention. Preliminary consultation with the main stakeholders involved is recognized as a fundamental step prior to the implementation of any innovative strategy. Without achieving such pilot work, there is a risk of jeopardizing the implementation of the intervention, thus reducing the possibility of finding any result at the evaluative phase.

This randomized trial assesses the effectiveness of an intervention using structures already in place to produce practice changes and combining simple realistic strategies of integrated care between primary care and oncology care that transfer into practice the main recommendations of expert groups and health authorities [22–25] to globally improve cancer supportive care.

To prevent confounding bias from variable organization of care, the study is restricted to a single center. However, if the intervention is proven effective, its essential conditions of success will be identified and could be replicated in other settings and extended to other cancer sites. The knowledge gained from the study findings may clearly enhance QoL for cancer patients and their FC.

## Trial status

Recruiting.

## Ethics approval and consent to participate

This study has received human ethics approval by the Institut Universitaire de Cardiologie et de Pneumologie de Québec (IUCPQ) Research Ethics Committee (Approval number: 2016-2523, 21206).

## Additional file

Additional file 1: SPIRIT Checklist. (DOC 122 kb)

## Abbreviations

CBS-EOLC: Caregiver Burden Scale in End of Life Care
ECOG: Eastern Collaborative Oncology Group
EORTC: European Organization for Research and Treatment of Cancer
ER: Emergency room
FC: Family caregiver
FP: Family physician
HADS: Hospital Anxiety Depression Scale
IUCPQ: Institut Universitaire de Cardiologie et de Pneumologie de Québec
ON: Oncology nurse
OPN: Oncology pivot nurse
OT: Oncology team
PDSQHS: Psychological Distress Scale used in the Quebec Health Survey
Pts: Patients
QLQ-C30: Quality of Life Core 30
QoL: Quality of life
